# Indigenous practices of environmental sustainability in the Tonga community of southern Zambia

**DOI:** 10.4102/jamba.v8i1.331

**Published:** 2016-11-03

**Authors:** Kennedy M. Kanene

**Affiliations:** 1 Department of Languages and Social Sciences Education, University of Botswana, Botswana

## Abstract

Culture plays a significant role in conserving the environment. The purpose of this study was to explore the measures that have been employed by the Tonga people of southern Zambia to sustain their local biophysical environment. The research focussed on investigating the strategies which they use to conserve the soil, water, animals, medicinal and fruit plants, and rangeland. A qualitative research design was used in the study. The data were collected through interviews with elderly Tonga people and herbalists, and through observation and personal participation in the daily life of the Tongas. The study reveals that selective harvesting, totemism and taboos, organic farming, crop rotation and intercropping, sacredness of water sources and traditional authority are the main instruments of environmental conservation amongst the Tonga. The article concludes that governments, policymakers and environmentalists need to give the conservation strategies employed by indigenous people the prominence they deserve for environmental sustainability.

## Introduction

There is a global concern to safeguard the increasingly dilapidating environment (Obiora & Emeka 2015). For example, the United Nations International Strategy for Disaster Reduction (2009) urges that environmental degradation needs to be combated to minimise or eradicate its threats to lessen the limit of the earth to meet social and environmental destinations, and needs. Anup (2005) says that, if not urgently addressed, the effects of environmental degradation can demolish the whole environment. Similarly, Kanene (2015) posits that the exploitation of our planet and degradation of our environment have gone up at an alarming rate such that concerted efforts must be applied to enhance environmental sustainability. The above issues require interdisciplinary and multidimensional approaches, which include the use of cultural construct, herein referred to as African indigenous knowledge system (Obiora & Emeka 2015). Ngara (2013) notes that indigenous knowledge is built by societies through generations, living in close contact with nature. This knowledge encompasses norms, a system of classification of natural resources, empirical observations about the local environment and a system of self-management that governs resource use. He further argues that traditional beliefs, cultural mores and practices are significant in the successful conservation of the natural environment. Culture has played a crucial role where environmental resources are under threat. Grenier (1998) adds that indigenous people with historical continuity of resource use practices often possess a broad knowledge base of the behaviour of complex ecological systems in their abilities.

As Lancaster and Vickery (2007) stated, the Tongas are believed to be the first Bantu-speaking people to live in Zambia; they have been living in southern Zambia since AD 1100. In addition, O’Brien and O’Brien (2007) further highlight that the Tongas are prominent agricultural people. They have lived sustainably within their biophysical environment for many centuries (Kaoma 2010). This is despite their being the leading agricultural ethnic group in Zambia (Kanene 2015). Moreover, United Nations Children’s Fund (UNICEF) (2013) has designated the Tongaland as a ‘No Open defecating Zone’ because generally all households possess a toilet or toilets. To this effect, bill boards are displayed in all main gateways to this area. Kaoma (2010) further reveals that the Tonga people are amongst the leading promoters of the concept of ‘going back to Eden’. The phrase entails that they more often than not run to herbal medicine for most of their health challenges. However, there is no known study that has been conducted to explore the strategies aiding them to live harmoniously with their environment. Therefore, the aim of this study was to unveil the practices and experiences that have enabled the Tonga people to live sustainably in their local biophysical environment for several years. The following sections provide some insights from the literature on how aspects of culture have helped conserve the environment in some parts of Africa.

## Culture and environmental sustainability

According to Lssozi (2012), African communities have rich environmental cultures which can be understood by listening to their myths, taboos, stories, proverbs and beliefs and also by observing their symbols and rituals. For example, amongst some indigenous African communities, the association of some animals and plants with spirits has enabled environmental sustainability (Forde 1998). Essentially, amongst the Igbos of Nigeria, there are taboos associated with some rivers and forests to safeguard them from pollution, abuse and exploitation (Obiora & Emeka 2015). There is also strict observation of moral order amongst traditional societies whereby people observe some ecological ethics such as not to defecate near streams where drinking water is fetched. It is believed that contempt to this prohibition attracts severe sanctions from the deity who owns the stream (Obiora & Emeka 2015).

The United Nations Conference on Environment and Development (UNCED) (1992) advocates for the restoration of the ethic of love and respect for the earth in people’s lives, which the traditional people have retained as central to their value system. This view is supported by Cooper and Palmer (1998), who say that indigenous people have lived in ‘oneness’ with their environment. These people live within the environment with an attitude of respect guided by their ecological wisdom (Cooper & Palmer 1998). Their sustainability practices are manifested through traditional modes of agriculture, namely, shifting cultivation, mixed farming and agroforestry (Wolfgram 2006).

The Ba’Aka pygmies of Central Africa represent a good case of indigenous people’s involvement in environmental sustainability. They are involved in managing flora and fauna in reserves. Their knowledge in conservation greatly contributes to the understanding of the links within the forest ecosystem, hence being invaluable for planning and management purposes (Ayong 2007; Lssozi 2012). They are cautious that few people hunt in one area at the same time for safety reasons; they also respect areas for trapping reserved for them in the forest and avoid use of guns for hunting to evade gun accidents which may result when too many hunters act too near to each other in the same forest. Also gun restriction, minimal trapping of animals during the rain season and sparing of young animals are meant to allow animal populations to increase. Furthermore, some sections of rivers or streams and forests are considered sacred; therefore, fishing and hunting are forbidden in these areas unless special rituals are to be performed. Also, to ensure harvest of wild nuts and fruits is not damaging to the plants, they grow fruit trees on their farms and around the village. In addition, they control poaching by assigning youth groups to guard the park to regularly monitor and report to village councils about the presence of poachers (Ayong 2007).

The Ba’Aka believe that they do not have to kill their totems for food because the reduction in such animals negatively affects the well-being of the people. It is also believed that hunting down of such animals is very difficult and would instigate some misfortunes to the society. In addition, pregnant women should not eat certain types of reptiles, birds and animals because eating them might stop production of breast milk or cause foetal abortions (Chikwanha 2011). In summary, the Ba’Aka case above simply illustrates the existence of viable environmental sustainability strategies amongst indigenous Africa communities.

### Description of the study area

The southern Province of Zambia is located between latitudes 15°S – 17°S and longitude 25°E – 29ºE. It receives an annual rainfall of 650 mm from October to March. Maximum temperature ranges from 15 ºC to 27 ºC in the cool season, with minimum temperature ranging from 6 ºC to 10 ºC and occasional frost on some nights in valleys. During the hot season, maximum temperature ranges from 27 ºC to 35 ºC. The mean annual temperature varies between 18 ºC and 20 °C. The highest annual average temperature is 32 ºC and the lowest average temperature is 4 ºC (Aregheore 2010).

The Tonga comprises about 89% of the population of the southern Province, and the remaining 11% is shared by the other 72 ethnic groups in the country (Central Statistical Office 2016).

## Methodology

This article resulted from the researcher’s long-term observation and inquiry of the life of the Tonga people regarding their harmonious interaction with the biophysical environment (Kanene 2015; Kaoma 2010). The study was basically ethnographic in nature, which took 15 years. ‘Ethnography’ means trying to understand the behaviour and culture by going out and talking to and observing people whatever they do (Ventres & Frankel 1996). As such, the study involved spending time with Tonga people as they went about their daily business in their natural setting to develop a better understanding of their indigenous environmental sustainability practices. The rationale for adopting the ethnographic design was threefold: firstly, to seek an in-depth understanding of how and why the Tonga conserve their local environment; secondly, the researcher wanted the community to appreciate that he was part of them so that they would respond with minimal suspicion to his questions on some aspects of conservation that needed clarification; and, thirdly, to actually practice some of the methods used by them to conserve the environment so as to experience how and whether they worked. The researcher viewed this approach as having less interference with the people’s lives as alluded to by Crabtree and Miller (1992). Essentially, the researcher was both a silent background observer and a ‘participant-observer’. In this regard, he simply silently noted what he observed about conservation (silent background observer) and asked questions for clarification as he accompanied the study participants in their daily activities such as farming, collection of herbal medicine, firewood, grass and water fetching (participant-observer) (Berkwits & Inui 1998). During such instances, the researcher talked to individuals for detailed understanding of topics that emerged from his observation.

The data for the study were gathered using unstructured observations and semi-structured interviews. Interviews were specifically conducted to seek explanation on general topics that he identified as he mingled with the community. Based on the emerging topics, some of the key interview questions included the following: ‘why does the Tongaland contain numerous indigenous fruit tree species and medicinal plants?’, ‘explain some of the sustainable farming techniques applied amongst the Tonga people, ‘what is the essence of totemism in the Tonga society?’ and ‘how have you ensured that there is viable rangeland in this animal husbandry community?’. The study involved interviews with 28 elderly (including traditional leaders) members of the Tonga society and 11 herbalists. They were purposively selected on the basis of their extensive and detailed knowledge of the Tonga conservation practices. In addition, the observation method was used to confirm some responses from the study participants about conservation and in identifying some of the environmental sustainability strategies that may not have been stated by the participants. Moreover, during the researcher’s long stay amongst the Tonga people, he participated in traditional farming as he owned about three hectares of farmland, and also used various traditional medicinal plants for curing common diseases such as malaria, dysentery, coughs, heartburns and others. Thematic analysis was employed for data analysis and the results of the study are presented in descriptive form.

## Results and discussion

### Conservation of plants

This section contains responses regarding strategies and the rationale for conserving plants amongst the Tonga people. The Tongaland hosts numerous indigenous fruit trees; according to most of the elderly Tonga people and indeed from the researcher’s observation, the fruits born from these trees include Masuku (*Uapaca kirkiana*), Mbula (*Parinari curatellifolia*), Nsombo (*Syzygium jambolanum*) Nchenje (*Diospyros kirkii*), Makunka (*Hyphaene ventricosa*), Makole (*Azanza garckeana*) Mfwulimuninga, Mawi, Maabo and Masau (*Ziziphus mauritiana*), Busiikka (*Tamarindus indica*), Mbubu (*Vangueriopsis lanciflora*), Fwutwe, Mang’ombyo and others. This is notwithstanding the fact that much of the land is cleared of forests for agricultural purposes. The researcher’s observation found that fruit trees are not only important as sources of food and as money earner through fruit sales but also serve as shade to the people during resting intervals as they work in their fields. They also serve as wind breakers and minimise the impact of storms on their crops. The Tonga people have generally ensured that fruit trees are left intact whilst other trees are cut to prepare fields for crops. Also, they habitually plant fruit trees such as mangoes along the boundaries and at the ends of fields. From the above discussion, it can be deduced that food value of a tree is one of the factors that inspires conservation. Ngara (2013) agrees with this assertion by stating that the worth of a tree to provide some food benefit is a significant motivation for the local people to conserve it.

Furthermore, all traditional leaders alluded that it is a taboo for Tonga people to use fruit trees for firewood. Similarly, Mapira (2013) reveals that in Zimbabwe some plant species were not used for firewood because of a belief that it would cause a lot of smoke – yet the idea was that they were fruit trees which did not need to be destroyed. Chikwanha (2011) also notes that the motivation to conserve these trees is that they provide extra benefits. This should guide environmental educators and conservationist on how to inspire people for conservation. The value of the trees should be clearly clarified to the masses so that they would appreciate their conservation.

Another noticeable feature in the Tongaland is the high presence of luxuriant big tree such as Mululwe (*Rauvolfia*), Mubanga (*Pericopsis*), Muzungula (Sausage tree), Mopane (*Colophospermum*), Mubombo (*Acacia*) and Mukuyu (*Ficus* *ingens*). Most of these trees are believed to be residents of the ancestral spirits. Observations during the study revealed that the bases of big trees with canopy for shade are used for social events and as groves for shrines, which is also supported by Nakashima (2009).

Amongst the Tongas, conventional medicine is more an alternative than mandatory. In the many years that the researcher interacted with the Tonga community, he discovered that they have high regard for traditional medicine, probably more than medicines from hospitals and clinics. It is no wonder when Mbiti (1969) says that you can take an African out of Africa but cannot take Africa out of an African. It was observed that, when troubled with much sickness, Tonga people would generally go back to traditional plant-based medicine.

In the extraction of nature-based medicines, they are usually concerned with environmental sustainability. The study found that for plant-root-based medicine, only parts of the roots should be cut from the plants. For some plants, only leaves should be used for medicine, whilst for plants whose backs were used, either the north and south or the east and west facing sides of the plants could have backs extracted. Most of the herbalists held that cutting all the roots, removing backs round the plant or cutting the entire plant for medicine would lead to the cutting (death) of the life of the patient. It is also believed that if one uproots a medicinal plant, that particular species would relocate to places far from the local community, thus being inaccessible and resulting in deaths locally. Similarly, Mapira (2013) found that in Zimbabwe medicinal plant species were believed of having a tendency to relocate because of abuse by the local community. However, further inquiry from the elderly and herbalists revealed that the extraction of only a few parts of a plant was tailored towards preserving its life as the plant would survive with the remaining parts. The above practice could be referred to as selective harvesting (Muhando 2005). It ensures that only parts of plants or animals whose removal will not retard regeneration and recovery of the affected plants, are extracted.

This researcher observed that, traditionally, the Tonga use wood fuel as their main source of energy. As such, it is demanded that only naturally dried parts of the plant should be extracted for firewood. In fact, most elderly members of the Tonga community noted that when collecting dry firewood, extreme carefulness should be exercised as certain trees were sacred and should not be used as fuel despite being dry. It was also found that only those species that were in abundance and were not fruit trees were generally considered for firewood. In addition, the researcher observed that it was customary for the Tonga to extinguish the fire immediately after use to conserve firewood.

Furthermore, some trees were believed to produce poisonous smoke, whilst some others were considered untouchable. According to the majority of the elderly respondents, touching such trees would cause terrible rush. Moreover, some plant species were not used as firewood because they were believed to instigate family conflicts. However, further inquiry revealed that such plants were initially amongst the threatened species. In this regard, most of the elderly study participants indicated that threats about their use had to be coined for conservation purposes.

### Conservation of animals and birds

An investigation about animals and birds conservation revealed that all the Tonga people belong to a particular clan. The clan names are associated with either fowls or animals as illustrated in Table 1.

**TABLE 1:** Clan names and associated totems.

Clan	Totem	Clan	Totem
Muleya	Goat	Mwiinde	Pigeon
Mudenda	Elephant	Muntanga	Antelope
Munsaje	Rabbit	Moono	Cattle
Mweetwa	Crocodile	Muzyamba	Elephant
Muloongo	Monkey	Munsaka	Dog
Muchindu	Lion	-	-

Accordingly, the Tonga regard the animal related to their clan as a totem. Both the traditional leaders and the elderly noted that it is taboo for any clan to eat their totem as they are perceived as sacred. Therefore, there is an intimate relationship between the totem and the clan to the extent that the clan does not eat, kill or trap these animals (Hens 2006). The researcher’s observations revealed that they also guard their totem against being killed by other clans who may not consider it as sacred. In the same manner, Mapira (2013) notes that totemism reduced hunting and gathering of some edible fauna and flora. Consequently, one of the largest national parks in the world, the Kafue National Park, is located in the Tongaland. Moreover, Lochnivar National Park that boasts of having the highest number of bird species in the world is also situated in the Tongaland (Kaoma 2010). Furthermore, the Tonga people own more than three quarters of livestock in Zambia (Central Statistical Office 2010). Essentially, totemism creates harmonious relationship between tribal groups and the natural environment (Grenier 1998).

Notably, Table 1 indicates that most of the totems are animals that are commonest and most vulnerable to extinction as they are easily accessible to man. Without culture-related conservation strategies, most of them would probably be extinct.

An observation of many species of scavenger birds and some rare birds prompted an inquiry. The study found that killing scavenger birds is a taboo amongst the Tonga. Culturally, such birds are never eaten, leading to their high population. These birds are extoled for cleaning the environment as they feast on carcases of cattle which die of foot and mouth disease almost annually. Moreover, according to most traditional leaders and a number of the elderly Tonga people interviewed, some birds are viewed to be kingly. Amongst such birds is one locally called nduba, a colourful and rare bird preserved for chiefs who sparingly use its feathers to decorate their regalia. The above respondents stated that it is abominable for commoners to kill such a bird without the permission of the chief. Moreover, the bird is also regarded as ‘lucky bird’ in that merely seeing it is believed to announce a fortune.

Furthermore, birds such as Tumba (owls) are regarded as ‘birds of bad omen’. Most elderly members amongst the Tonga stressed that killing them is a taboo followed by a bad omen to the offenders or their family. They are conserved for their role in preying on crop pests, especially rats. This is supported by Kanene (2015) who posits that owls are famous for checking the increase in the population of rats in the Tongaland.

Further observations discovered that the Tonga culture prohibits pregnant women from eating eggs. It is widely believed that breaking this regulation would cause the woman to give birth to a child who may never have or at least may delay to develop hair on his or her head in resemblance to hairless eggs. In-depth probing amongst the elderly respondents indicated the underlying rationale of the prohibition as being that permitting pregnant women freedom to consume eggs would probably lead to the extinction of fowls such as chickens as pregnant women tend to be very obsessed with certain nice foods, eggs inclusive.

### Agricultural conservation practices

This section reveals findings about how Tongas practice sustainable agriculture. According to the Central Statistical Office (2016), the Tonga are the chief producers of Zambia’s core food crops. Consequently, much of the maize exported by the country either comes from Tongaland or is produced by this ethnic group in many areas of the country where they are scattered (Kanene 2015). For many centuries, they have continuously produced various crops in their land. The study revealed application of environmentally friendly practices as the basis of sustaining their soils for agriculture. The observations by the researcher revealed that, commonly, Tonga people use crop rotation in their farming essentially involving maize, sunflower, groundnuts, soya, beans, peas, sorghum and sweet potatoes. Maize is grown in the same field for about 3 years before another crop is planted. This benefits the land through the difference in crop nutrients requirements and different nutrients fixing in the soil. In addition, this helps break disease cycles of crops. Also, it helps maintain the viability of the land for nutrient availability (Kanene 2015).

The study also revealed intercropping as being an important practice of the Tonga. The researcher observed that within the same field it was common to find at least three different crops. For example, a maize field could be intercropped with pumpkins, groundnuts, cucumbers, watermelons and sweet grass. A groundnut field could be intercropped with cucumbers, watermelons and sweet grass, whilst a beans field is usually intercropped with cucumbers, watermelons, sweet grass and a bit of maize. Apart from the benefit of the soil being sustained for nutrients, intercropping provides a variety of nutrition to the community, as different crops with varying nutritional values are grown in one field simultaneously. Moreover, it is a crop failure mitigation factor which ensures the survival of some crops if others fail because crops vary in terms of growing conditions. Besides, intercropping could be credited for the Tonga region (southern Province of Zambia) being least in terms of hunger in the country despite being drought prone. Also the fact that intercropping conserves the soil, reduces pests and increases crop yield, was revealed. In this regard, Chapungu (2000) posits that intercropping can reduce pest damage by 80%.

It was further found that environmental sustainability has been widely practiced by the Tonga people in their treatment of the land. In his long stay and participation in agriculture amongst the Tonga people, the researcher witnessed widespread use of livestock manure to sustain land. In this case, usually grass and crop residues are added in cattle kraals to add bulk to the amount of organic remains. Between August and September, they dig out and ferry the manure to their fields. Arguably, organic manure helps repair soil texture, recovery of soil nutrients and is environmentally friendly (Chikanhwa 2011).

The other common soil preservation practice is agroforestry. Generally, in every field the researcher noticed trees such as Musangu and Muunga. Most of the elderly people pointed out that the presence of such trees indicates fertile soils; therefore, they are either maintained or planted. It was revealed that the plants can transform poor soils into fertile ones in the long run. The elderly respondents further disclosed Bbondwe and Mutandazyeelo as being amongst the other plant species that add to soil fertility. As such, some sections of the Tonga propagate seeds of these weeds on their land to improve soil fertility.

It was also observed that, largely, the Tonga do not burn crop residues. Much of the residues are left in the field for organic manure. In addition, most farmers having demolished anthills apply the soils in their fields to add nutrients. Essentially, Lssozi (2012) notes that organic farming has helped indigenous people sustain their soils and hence reviving this practice would help in soil water conservation, mitigating climate change and ensuring sustained biodiversity.

Another notable agricultural strategy amongst the Tonga people is fallowing. It was observed that the land is left unploughed for a number of years, at least four years, to enable it to regenerate. A year before crops are planted, the field is ploughed without planting anything on it. Commenting on this phenomenon, Mushuku *et al*. (2014) argue that fallowing regenerates the physical and chemical properties of the soil, suppresses weeds, pests and disease, and reduces soil erosion and runoff. The other noticeable form of farming in the Tongaland is zero tillage. In this regard, some holes are dug where seeds are planted leaving the other areas intact. This conserves soil structure and fertility as well as reduces soil erosion (Chapungu 2000).

It is also worth noting that most indigenous Tonga people respect planting of indigenous seeds. In the observation of the author, and indeed through the testimony of most of the elderly respondents, the best of the grain seeds are preserved by hanging or stacking them in the roof of the kitchen where smoke acts as grain preservative against pests. Besides, some of the seeds having been thoroughly mixed with ash are deposited in an airtight container which is then kept in a cool room until planting time. As for sweet potato seeds, the researcher observed that the farmers leave some of the potatoes in the soil during harvesting to germinate with the first rains and are allowed to mature for planting. Also, some creepers are cut at harvesting and then planted in gardens to be watered till the subsequent season.

### Water conservation

An investigation on water sustainability established that the Tonga regard some water sources to be sacred. In this case, sacredness is associated with either the whole or part of the water source. Usually, the water sources are perceived as habitats of gods such that some tend to be upgraded into shrines. Most of the traditional leaders together with the elderly participants in this research held that during seasons of drought or delay in the onset of rains, the village elders visit the shrines to pray for rains. Because of a common belief that rain spirits abide in these areas, the water sources are kept ‘pure’. The majority of the research participants indicated that purity entails no bathing, washing or dumping of refuse in the water. Similarly, Garutsa (2014) found that the Eastern Cape pools were considered abodes for mermaids; thus, people were discouraged to venture in such areas. Violating this norm attracted sanctions by spirits and punishment by traditional leaders (Garutsa 2014). In fact, traditional norms also discourage the unwarranted destruction of wetlands. They are a source of water during drought as communities sink traditional wells in wetlands.

It is also a custom of the Tonga not to have crop fields along water courses or dam catchment areas. According to most of the elderly and traditional leaders, it was believed that eroded soils from the fields would increase siltation, resulting in the ‘death’ of these water sources. In addition, they conserve vegetation along riverbanks. According to Osunade (1994), these practices not only protect water catchment areas but also serve as measures for minimising flooding and soil erosion.

Furthermore, it is prohibited to defecate in open areas within the catchment area of the water source. The study revealed that defecating must be done in a toilet or a hole dug in the ground which must be buried after use. Likewise, amongst the Marakwets, Lssozi (2012) found that it was a taboo to wash in or near a water source. Moreover, lactating mothers are restricted from water points; throwing objects into watersheds is also prohibited. Similarly, amongst the Tonga, it is forbidden to wash clothes near water collection points. In fact, the Akans of Ghana also forbid discharge of industrial and human waste into water bodies lest the culprit is punished by the gods or traditional authorities. Customary laws mandate users to keep lakes, rivers and other water sources pure as they are regarded as dwelling place of gods (Cooper & Palmer 1998; Lssozi 2012).

### Grass conservation

Grass is an important resource amongst the Tonga; hence, measures are taken to ensure its sustainability. Findings from most of the traditional leaders, the elderly and the researcher’s observations all agree that before the annual cutting of grass by the Tonga people, commissioning by traditional authorities has to first occur to certify that grass has matured enough to shade seeds. In addition, during harvest, seeds are shaken off to facilitate germination of fresh grass. Ngara (2013) had a similar finding in Botswana that grass for thatching traditional houses is harvested in winter because by that time seeds are ripe enough to fall off for growth in the rainy season. Besides, sickles used for grass harvesting do not affect the roots, thus ensuring sustainable grass growth.

Furthermore, it was observed that the Tonga practice an annual (around August) burning of grass in their rangeland. They believe that burning ensures good growth of fresh pasture and kills animal pests including ticks. Besides, early burning prevents soil erosion and ensures that the period for reduced pasture for their livestock is reasonably short to the early rains. To protect some areas from the fire, they prepare fireguards which are primarily done by using fire itself. They burn along the boundary of the intended pasture land before burning the targeted pasture area. Burning often occurs in the evening when the wind speed has generally subsided for easier fire management.

## Conclusion

Indigenous people have contributed quite immensely in conserving the biophysical environment. The Tonga people have conserved animals through use of totems, vegetation via taboos and the land through organic farming, fallowing and crop rotation. Also the attribution of sacredness and prohibition to defecate close to water sources, keeping river catchment areas vegetated and avoiding having crop fields close to water sources have been used to conserve water. Wild fruit trees have been preserved by sparing them when clearing vegetation for various development purposes. In addition, some indigenous trees are considered sacred, whilst others are used for social event and as land boundary markers, and medicinal plants have been conserved by selective harvesting.

The Tongas’ environmental sustainability strategies have been proved very practicable; thus, for effectiveness, efforts on biodiversity conservation can learn from their context-specific local knowledge and institutional mechanisms. This is because the Tonga have cultivated and used biodiversity sustainably for centuries by supporting maintenance of healthy ecosystems (United Nations [UN] 2013). Therefore, conservationists should ensure that they incorporate indigenous people’s practices in environmental conservation. Governments and policymakers should also integrate indigenous knowledge into environmental policies and take advantage of this knowledge to minimise environmental degradation (UN 2013). Finally, I would appeal for genuine partnership regarding scientific and indigenous knowledge towards environmental sustainability.
